# Accessibility in People with Disabilities in Primary Healthcare Centers: A Dimension of the Quality of Care

**DOI:** 10.3390/ijerph191912439

**Published:** 2022-09-29

**Authors:** Maggie Campillay-Campillay, Ana Calle-Carrasco, Pablo Dubo, Jorge Moraga-Rodríguez, Juan Coss-Mandiola, Jairo Vanegas-López, Alejandra Rojas, Raúl Carrasco

**Affiliations:** 1 Departamento de Enfermería, Facultad de Ciencias de la Salud, Universidad de Atacama, Copiapó 7500015, Chile; 2 Departamento de Kinesiología, Facultad de Ciencias de la Salud, Universidad de Atacama, Copiapó 7500015, Chile; 3 Programa de Magíster en Metodología de Investigación Cualitativa para la Salud, Facultad de Ciencias de la Salud, Universidad de Atacama, Copiapó 7500015, Chile; 4 Escuela de Obstetricia y Puericultura, Facultad de Ciencias Médicas, Universidad de Santiago de Chile (USACH), Santiago 8320096, Chile; 5 Facultad de Ingeniería y Negocios, Univerdidad de Las Américas, Santiago 3981000, Chile

**Keywords:** access to health services, people with disabilities, primary healthcare, quality control, right to health, universal accessibility

## Abstract

The purpose of this research is to evaluate universal accessibility in primary healthcare (PHC) centers in the Atacama region, Chile, through an analytical cross-sectional study with a quality approach, which uses the external audit model with the application of a dichotomous comparison guideline, evaluating levels of compliance with four dimensions of universal accessibility described in the literature: participation, information, accessibility chain and architectural aspects. This was carried out in 18 PHC, and set as Lower Control Limit (LCL) of 70% to compare levels of compliance, and a hierarchical model and k-mean analysis were applied. Results: Very low compliance averages were obtained, 37.7% participation, 4% information, 44.4% access chain, and 63.9% architectural aspects, indicating a critical situation. Moreover, the cluster comparison allowed to observe that a group of healthcare centers complies more than other groups, which requires more attention. Conclusions: The low level of accessibility for people with disabilities may be associated with various factors that require further monitoring and analysis. However, low levels of accessibility require changing the way of relating to this vulnerable group of the population, and considering including them in the design and reasonable adjustments made in PHC centers. The findings from this research open the possibility for future research that increases understanding of how to reduce barriers in a such wide variety of forms of disability.

## 1. Introduction

It is estimated worldwide that 15% of the population live with some kind of disability, this being more prevalent in impoverished or developing countries. In Chile, this figure reaches values of 20% [1]. This association between poverty and disability is expressed in a bi-directional way [2,3,4], causing people with disabilities to have less economic income, a lower level of education, a lower employment rate, and a greater demand for medical care [3,5]. People with disabilities are considered a vulnerable group due to the health risks related to their functional condition and due to the multidimensional poverty context in which they find themselves. This can be observed even in developed countries, such as Canada and the United Kingdom that have universal access to health but that, in the same way, describe barriers that affect the accessibility of people with disabilities [6]. In this sense, there is a notorious inequality between people with and without disabilities, as the former must face multiple barriers to access health services [7,8].

The literature on care for people with disabilities suggests that they face various obstacles to access health services, suffering from discrimination and less access to preventive benefits than people without disabilities [9,10,11]. In this regard, the World Health Organization (WHO) [12] highlights barriers linked to attitude issues, physical barriers, barriers that hinder communication and economic barriers. Research in the US and Australia, where there is no universal access to health, reported that people with disabilities are mostly limited in access to primary healthcare (PHC) not only due to environmental barriers, but also due to their lower ability to pay [13,14].

The gap in access to care for people with disabilities is therefore a global problem of which impoverished and developing countries are just becoming aware to address it from public policies due to the increase in disability and higher life expectations of the population [15,16,17].

Improving accessibility to primary health care should be a priority for all countries in the world, considering that health is a fundamental human right [18]. We must not forget that the Convention on the Rights of People with Disabilities and Optional Protocol, of the United Nations (UN), in its article 25, states that “the States Parties recognize that people with disabilities have the right to enjoy highest attainable standard of health without discrimination on the basis of disability”. States parties shall take appropriate measures to ensure access for people with disabilities to health services that consider gender issues, including health-related rehabilitation. Therefore, actions must be committed by the states to favor the reduction in gaps in access to health for people with disabilities [19], contributing to greater equity since these people require more health care and, on the contrary, they find greater access restrictions [17,20]. From this perspective, universal access constitutes a challenge for health systems, as it implies generating innovations to traditional actions, ensuring the necessary resources to provide efficient and sustainable care over time [20,21,22,23,24]. In this sense, health systems play an active role in providing timely and permanent care for all people [25].

Báscolo, Houghton and Del Riego [18] evaluated access and coverage in five Latin American countries that promoted health reforms focused on innovations in insurance and care models for the population in conditions of social exclusion. In this study, primary health care models with a comprehensive perspective prevailed. Although these models showed different results in the countries evaluated, in Chile, Mexico, Salvador, Peru and Uruguay, progress was recognized in access and care for economically less favored people, an important fact for health systems without universal access to care. Chile, in an effort to improve accessibility, implemented the explicit health guarantee system, which has been one of the most relevant aspects of the public policy, contributing to reducing equity gaps, but with certain limitations [26].

The concept of accessibility understood as “the degree of adjustment between the characteristics of the population and the healthcare resources” corresponds to a dimension of access [27], while universal design or design for all is committed to social justice in favor of social and health services accessible to all, allowing everyone to live a life with dignity [28]. Universal accessibility should not be seen as an ideal but as a real aspiration so that people with disabilities can demand the right to fundamental services for their legitimate development.

This will depend to a large extent on the adjustments made to the infrastructure, processes and attitudes of health personnel [29], so identifying gaps in healthcare contributes to improving coverage and access to the vulnerable population [26,30] and evaluating public policies enacted to ensure the fundamental rights of this particular group of people [5,29,31,32].

### National and International Universal Accessibility Regulations

Assessing universal accessibility contributes to diversity, reduces disparity in health and contributes to equity [33]. From this perspective, Chile approved the National Technical Standard NCh3267:2012 [34] management systems for the inclusion of people with disabilities in 2012 and updated it in 2021. It is based on the Spanish standard UNE 170001 2:2007 [35] and was submitted to a national technical committee of experts on accessibility, to establish the requirements that a management system for the inclusion of people with disabilities must meet. For this reason, the regulation requires a policy of inclusion of people with disabilities in all public and private institutions with public access, and the creation of procedures to carry out a diagnosis of inclusion as a basis for planning continuous improvements to inclusion and universal access of people with disabilities [36]. The criteria established in the standard respond to the ambulation, apprehension, location and communication (DALCO) NCh3271:2012 criteria, which establish criteria for global accessibility, referring to the environment and accessibility conditions in terms of the different activities that people commonly carry out, to wander, communicate, reach, understand, use, and manipulate [34].

Although this standard does not provide uniformity, it constitutes a model that serves as a reference to develop a management system that responds to an institutional policy to include everyone. For this purpose, a management system is defined as “a set of mutually related elements or that interact to establish the policy and objectives, in addition to directing and controlling an organization aimed at ensuring rights and eliminating any form of discrimination based on disability” [36]. The use of a monitoring system does not respond to the use of global indicators since these are related to socio-cultural differences between countries and the relevance of promulgated regulations [5,37].

In the context of primary care, the literature suggests the need to build services according to the needs of people with disabilities [38]. At the national level, there are no studies that describe the levels of compliance with universal accessibility criteria in PHC centers. In this regard, in the comprehensive healthcare model (MAIS) promulgated in the health reform in Chile in 2005, the state undertook to “facilitate and ensure access to care for the most vulnerable groups” [39]. However, the evident lack of monitoring of universal accessibility can affect people who, due to their disability, are excluded from access to PHC.

The literature on accessibility monitoring for people with disabilities at a global level is scarce, and contrasts with the large amount of literature that exists on disability. However, interest in accessibility to primary health care is an increasingly relevant issue for disability researchers. The review carried out by Groenewegen et al. [40] in 31 mainly European countries described a great variability in the accessibility of people with physical disabilities to primary care, demonstrating that the issue is still a challenge at the level of public policies for countries.

The purpose of this research is to evaluate universal accessibility for people with disabilities in PHC centers through a pilot monitoring project carried out in the Atacama region, Chile. It is considered a contribution to the rights of people with disabilities, by showing critical nodes to primary care teams and political decision makers to consider future and possible strategies for continuous improvements to accessibility in PHC centers.

## 2. Materials and Methods

### 2.1. Data Collection

The study design is analytical cross sectional. Eighteen healthcare centers were evaluated, which corresponds to 95% of the universe of existing primary care centers in the region. The data were collected in the field through the verification of standards described in a dichotomous comparison guideline, which considered the existence or not of the evaluated criterion. These guidelines were built by the researchers based on the National Technical Standard NCh3267:2012 [34], management systems for the inclusion of people with disabilities, dalco Standards CCh3271:2012 [41], and the guidelines participation techniques in health established by the Chilean Ministry of Health [39]. This considered four dimensions that measure accessibility quality: (i) participation, (ii) information, (iii) accessibility chain and (iv) other architectural aspects. In total, the instrument contains 34 criteria or standards necessary to measure the quality of universal accessibility (Table 1). The dimensions were contrasted with a literature review that agreed on the importance of these quality dimensions to comply with universal accessibility in health centers [6,22,23,42,43]. The checklist weighted all the included criteria equally, without considering one aspect of another essential since it corresponds to an exploratory phase for the construction of an instrument that later establishes the essential criteria for people with disabilities [44].

The techniques of observation and verification of compliance with technical criteria were used: taking measurements using a tape measure, simulating a person with a disability using a wheelchair to make the routes, checking flows and spaces in situ, photographs of plans and checking compliance with standards, following the access chain from the transport stops public to the health center. The inspections were carried out by two evaluators per center, and the observations obtained were compared independently. One of the evaluators was a principal investigator, while the other was a professional engineer expert in risk prevention. This evaluation system corresponds to a quality audit system through external parties to maintain high criteria of objectivity [45].

### 2.2. Analysis Methods

For the analysis of the results, a hierarchical model and k-mean analysis were applied, considered classification and data mining methods. These allowed the classification into k groups determined by a close mean value that allows the different groupings observed.

The results with Lower Control Limit (LCL) of 70% were described, considering greater than or equal to 95% is very good, [80%,95%) is good, [70%,80%) is fair, and less than 70% is critical (non-compliant) [46].

**Figure 1:**
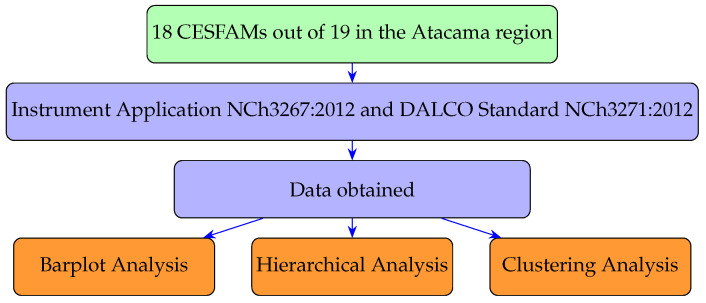
Research flow chart.

Figure 1 shows the research process from the selection of the Family Healthcare Center (CESFAM) in the Atacama region, the application of the NCh3267:2012 instrument and dalco NCh3271:2012 standard, and the data obtained up to the different analysis methods such as bar graph analysis, hierarchical analysis [47] and clustering analysis (k-means) [48].

The software R version 4.2.1 (R Core Team: Vienna, Austria) was used in this research work as a statistical analysis tool [49].

## 3. Results

The Atacama region has 19 centers for a population in 2017 of 286,168 inhabitants [50], and it corresponds to one of the 16 regions that make up the Chilean territory. According to the National Disability Study in 2015, the general prevalence of disability in Atacama reached 16.4%, while in the adult population it was 20.01%, being very similar to the national prevalence [1]. Eighteen health centers were evaluated following the comparison pattern that faithfully replicated the procedure since the same evaluators were always maintained, covering 95% of the regional geographic area. The conditions of the centers were verified in the field, disregarding the date of construction, given that, according to the current legislation, health centers must comply with accessibility standards and, if this is not possible, make reasonable adjustments. The main limitation when collecting the data is related to the verification of standards, which only give the possibility of establishing whether a center complies or does not comply, for what intermediate efforts are made invisible in this study.

**Figure 2:**
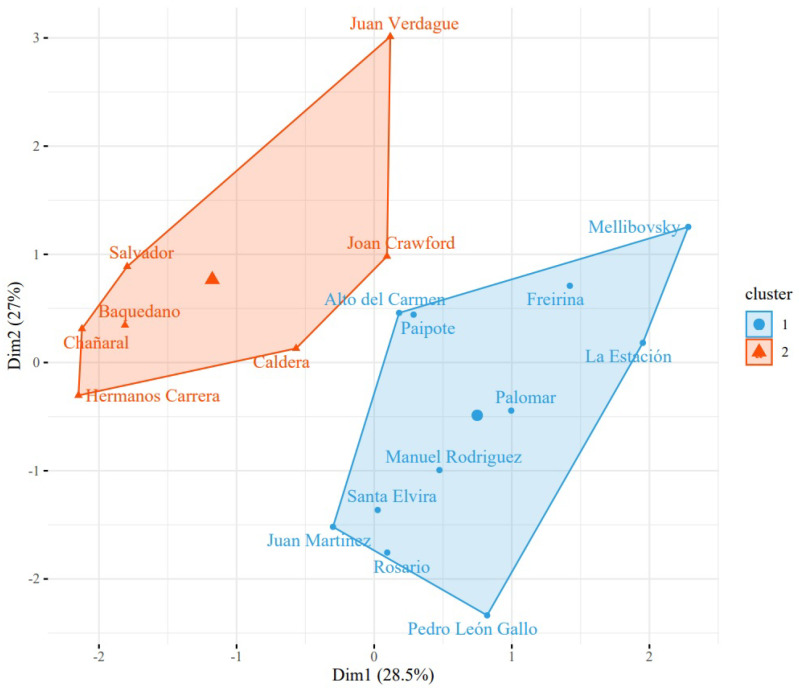
Two groups of primary health centers using k-means.

**Figure 3:**
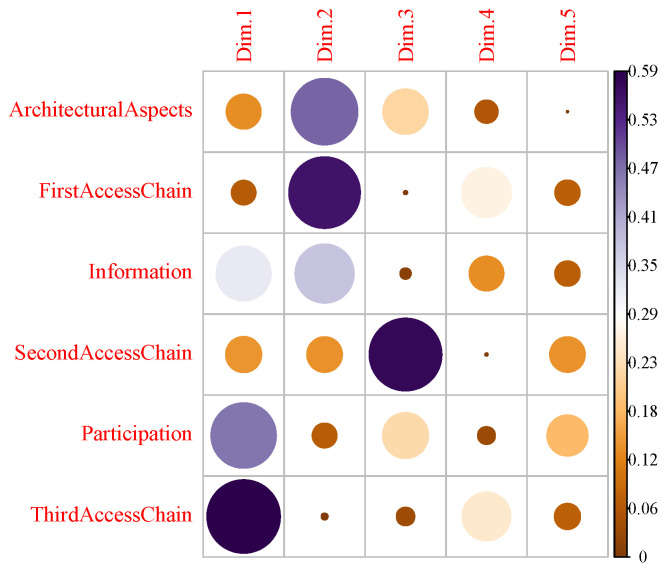
Cos2 variables.

At a general level, the critical state of universal accessibility is highlighted since a total average compliance of dimensions of 39.8% of the evaluated standards is obtained. It is noteworthy that the lowest dimension is related to the information dimension, reaching only 4% compliance, which implies that it is the most neglected dimension (Table 2). On the other hand, when performing a k-means as a grouping method based on the measurements in Table 2, it can be seen in Figure 2 two groups of primary health centers, where the red group represents the health centers that are below the average of general compliance and where the k-means technique is used to generate these two groups seen from the hyperplanes represented by Dimensions 1 and 2. They are mainly represented by the variables participation, third access chain and information in Dimension 1, and architectural aspects, first access chain and information in Dimension 2 as can be seen in Figure 3. In addition, in Figure 2, it is observed that Dimension 1 represents 28.5% of the system information and Dimension 2 represents 27%.

**Figure 4:**
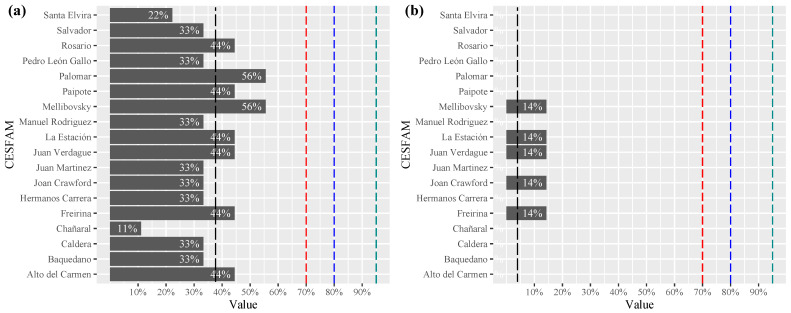
Level of compliance with the (**a**) participation dimension, (**b**) information dimension. (Family Healthcare Center (CESFAM), mean dash **– –** black, lim 70% dash **– –**red, 80% dash **– –**blue and 95% dash **– –**green).

In the dimension participation of people with disabilities, it is observed that the level of compliance of PHC centers reaches an average of 37.7%, with a dispersion contained between 11.1% and 55.6%. The centers that obtained the highest level of compliance were located in the same commune and belonged to different periods of architectural construction (see Figure 4a). The subdimensions evaluated considered the following: participation in civil society organizations, representation in participation activities in the healthcare center and the existence of registries of organizations of people with disabilities, among others.

In the information dimension, the communication procedures that have been established for people with sensory and cognitive disabilities (Chilean sign language, braille, audio and printed material) were evaluated (see Figure 4b). The average compliance of healthcare centers was only 4%, being the least addressed dimension despite being considered key in the management of universal accessibility.

**Figure 5:**
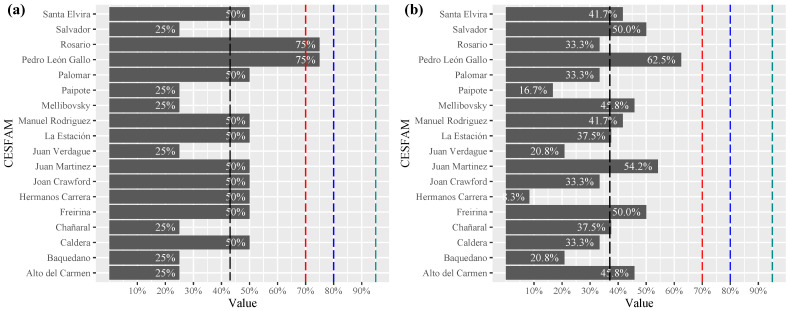
Level of compliance with the (**a**) first access chain, (**b**) second access chain. (Family Healthcare Center (CESFAM), mean dash **– –** black, lim 70% dash **– –**red, 80% dash **– –**blue and 95% dash **– –**green).

In the accessibility chain, the first contact assessed access to public transport to geographically access the health center (see Figure 5a). The cumulative average compliance of the healthcare centers was only 43.1%, with a compliance interval between 25% and 75%. Two centers stood out with 75% and 77% compliance, they had at least two public transport systems that reached the health center. On the other hand, 94.4% of the centers did not have updated information on the available transport lines that allow the beneficiary population in a situation of disability to be informed about the means that allow them to travel to the center. In most cases, there were no public transport stops in the surroundings, and when one was found, it did not comply with the universal accessibility standards.

The second contact evaluated the main access to the center, considering doors, ramps, railings and parking lots (see Figure 5b). The center with the best level of compliance only met 62.5% of these criteria. Only 20.4% of the centers complied with having a parking lot for people with disabilities, which must be accessible, safe and with good visibility. Main access with ramp at the entrance and exit was verified in 94.4% of the centers. However, ramps with different inclinations were observed that did not meet the degrees required by the current standard; some were very high and others had a downward slope, with the serious risks and difficulties that this implies for those who use them.

**Figure 6:**
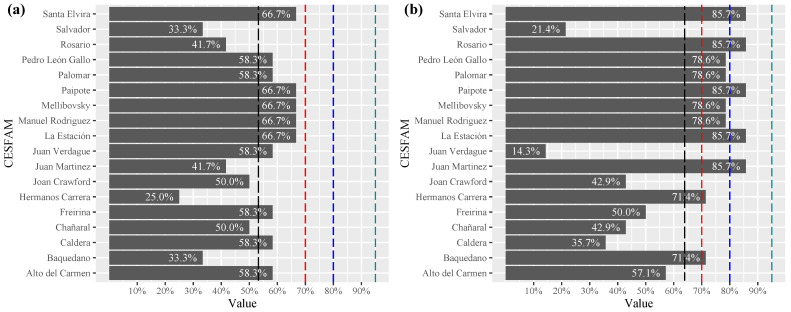
Level of compliance with the (**a**) third access chain, (**b**) architectural. (Family Healthcare Center (CESFAM), mean dash **– –** black, lim 70% dash **– –**red, 80% dash **– –**blue and 95% dash **– –**green).

The average compliance regarding the third contact involves the infrastructure of the Medical and Statistic Orientation Service (SOME) (in Spanish “Servicio de Orientación Médica y Estadística”) (see Figure 6a). In this case, special adjustments are required to serve people who use technical aids, such as canes and wheelchairs. The height of the dispenser on duty, height of the counter, depth of the tables to accommodate a wheelchair and communication processes for public attention with different needs were considered, reaching only an average of 53.2%. It was found that it is common to leave furniture and other goods in circulation corridors that constitute physical barriers that reduce spatial flow and increase the risk of accidents for people.

The average compliance of health centers regarding architectural aspects was 63.9%. It is important to note that 11 of the centers achieved 70% compliance, whereas one of the centers reached only 20%, well below the average achieved in the region (see Figure 6b). Among the criteria with the highest compliance were the standards for the use of stairs, use of railings, spaces between steps, use of shadows and non-slip floors, among others. In addition, only 44% of the centers complied with the presence of lever door handles, which are used so that people with neurological damage can push the door with their elbows or another part of the body, thus providing them with autonomy of access.

**Figure 7:**
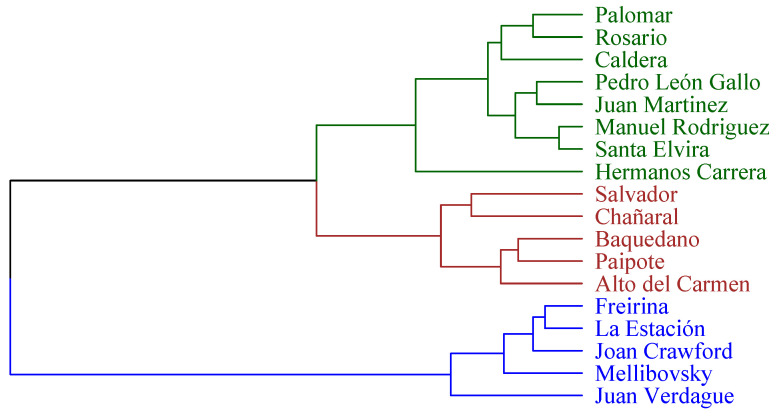
Hierarchical analysis: —highest compliance in the dimensions of the access chain and infrastructure, —lowest total compliance, —highest compliance in the participation and information dimensions.

Figure 7 allows visualizing different conglomerates of PHC centers, where the blue group represents the centers with the highest compliance, especially in the participation and information dimensions. On the other hand, green groups the centers with the highest compliance specifically in the dimensions of the access chain and infrastructure. Finally, the centers in red color represent the centers with the lowest total compliance.

## 4. Discussion

The results of this study revealed very insufficient levels of compliance in the dimensions evaluated. This reinforces the idea of how difficult it is to implement public policies in favor of people with disabilities, coinciding with the global trend. Adugna et al. [51], in a systematic review on barriers to health services in children with physical disabilities from African countries, described a series of structural factors that affect the implementation of services according to the specific needs of people with disabilities, thus limiting their access to basic health services and rehabilitation. A range of research works agree on the existence of architectural barriers, in accessibility chain, difficulties in public transport, and geographical barriers in healthcare centers, which is aggravated when people live in rural areas [16,32,52,53].

The results cannot be compared at the national level since there is no previous history of accessibility in PHC centers. According to the results obtained, the dimension of other architectural aspects (63.9%) obtained better levels of compliance than the other three dimensions evaluated, a fact that is explained by construction regulations that have progressively included different adaptations in favor of universal design [54]. In this dimension, the structural adaptations that have been part of the design or the modifications that were carried out within the health centers were considered, which allows to facilitate the movement and particular needs of people with disabilities, and they are found in the form of handles, handrails, universal bathroom, ramps, stairs with shadows, floor texture, and furniture heights, among others [55].

The accessibility chain dimension had an average compliance level of 44.4%. This refers to the aspects of the displacement that people with disabilities make since they leave their homes, use public transport, arrive at the healthcare center, enter, receive care and then return to their homes, having also received information and safeguards on their security [56]. The low level reached by the centers coincides with that reported in various countries, where there are multiple barriers in the environment that limit accessibility to health for people with disabilities, thus violating their right to health [22,25,51,53]. From this perspective, the un [57] committed its member states to accelerate the agenda for issues related to the inclusion of people with disabilities. Some active strategies implemented to improve access to healthcare services include the provision of subsidies for emergency transportation, work with leaders of community organizations, dissemination of success stories of inclusion, and conversations to raise awareness among the population about the stigma of disability [32,58].

The participation dimension of people with disabilities reached 37.7%, a worrying fact, as participation is a fundamental element in processes of social transformation. The Convention on the Rights of Persons with Disabilities states in Article No. 29 [59] that states shall guarantee people with disabilities political rights and the possibility of enjoying them on equal terms, committing themselves to actively promoting an environment in which people with disabilities can fully participate, associate in activities related to public and political life, and form organizations that represent them [36]. This right to participate is transversally integrated from public policy toward the execution of programs without discrimination. In this sense, it is the responsibility of PHC to act actively to strengthen community participation, responding to principles of governance and local development [60]. The study by Rezapour et al. [61] showed that people with disabilities consider governance as an essential issue in the quality management of primary care facilities.

The information dimension was almost imperceptible with a 4% of compliance. It constitutes a barrier that affects the possibility for people with disabilities to exercise their autonomy and benefit from health advances. This aspect was considered in Chile with Law 20584 [62], which regulates rights and duties that people have in relation to actions related to their health care. Providers were obliged to comply with the delivery of information adapted and understandable to any condition of people in order to achieve adequate guidance. Communication barriers affect the decisions that are related to the lives of people with disabilities, denying them the right to seek, receive and impart information and ideas on an equal basis with other people. In this regard, adapted information is key to people with disabilities can consent about health procedures [63].

The main difficulty in managing information in health centers is having suitable personnel to collect, safeguard and deliver the adapted information, especially when patients suffer a sensory disability [64,65,66,67]. The way in which health personnel handle information with users can become a facilitator or a barrier to accessibility. Aspects such as training healthcare personnel and maintaining a good relationship and attitude with patients have been described as facilitators of communication [68]. In this sense, the study by De Oliveira et al. [7], in Brazil, states that people with sensory disabilities are not considered in the planning of services, limiting their access to health and life in society.

Arguably, moving toward a developed society requires more just, equitable and inclusive public policies, and it is the responsibility of the state and society to make changes that favor the inclusion of people with disabilities [16,63]. The direct costs of lack of accessibility translate into opportunity costs and lost health benefits for this particular group. A person with a disability who cannot access information, transportation or a building creates a disadvantage that implies a loss of freedom when choosing and accessing a service, or participating socially [69], discouraging the use of PHC services, as mentioned by Bailey et al. [70] for bad experiences, as people cannot always respond resiliently to mistreatment.

Inclusive development requires permanent efforts so that people with disabilities can benefit, as well as the rest of the population, from production advances and services. In this sense, the un Committee on the Rights of People with Disabilities [57] has recommended at the national level that accessibility be included in transport, buildings and public facilities, information, and communication, both in cities and in towns of rural areas, with specific deadlines and sanctions for non-compliance, where organizations of people with disabilities are involved in all stages of their development, especially in monitoring compliance. The findings of the study make it possible to make visible that, despite the legislative advances in the country and Latin America, it continues to be a pending issue in PHC.

## 5. Conclusions

This study was able to establish that healthcare centers in the Atacama Region have insufficient levels of compliance to allow universal accessibility and free access for people with disabilities. This reflects what is happening at the national level. The most invisible item is information and in second place, participation, while architectural aspects and access chain obtain higher levels of compliance. To this point, the implementation of mandatory public building policies with higher standards in universal accessibility has contributed.

We propose to implement a monitoring system for universal accessibility to primary care centers at the national level, which would contribute to detecting barriers and promoting strategies that facilitate reasonable adjustments for the greater inclusion of people with disabilities: key aspects in the processes of improving accessibility, and empowerment of people in the exercise of their right to health.

Adjustments to eliminate attitudinal barriers require greater attention through training for personnel that must include specific characteristics of people with disabilities, incorporation of Chilean sign language and Braille, information in different formats and orientation to users considering the wide diversity of people with disabilities.

Finally, it is the health institutions that are responsible for adapting their care systems to the needs of people with disabilities.

The main limitation when collecting the data is related to the verification of standards, which only allowed the possibility of establishing whether a center complies or does not comply, so intermediate efforts were made invisible in this study.

## Figures and Tables

**Table 1:** Criteria evaluated in the universal access comparison guideline in PHC centers.

Criteria	Description	Standards
Participation	People with disabilities and their families are co-responsible for making diagnoses, advising, and proposing improvement measures in the health center, in order to promote universal access for all.	It measures nine criteria: existence of a diagnosis on the situation of universal accessibility in the center, participation in a consultative or decision-making council for people with disabilities, priority on waiting lists, monitoring of access barriers, participation in the regional disability table, design of duties and rights letter adapted to people with disabilities, system of claims, suggestions and congratulations adapted to people with disabilities, and existence of a facilitator for communication with deaf people, among others.
Information	Creation of systems that facilitate access to communication for people with disabilities, especially those with sensory or cognitive difficulties.	It measures seven criteria; the available information is in Chilean sign language, Braille, and in various formats such as written, video and audio, and also considers the training of staff to communicate assertively with people with disabilities.
Access Chain	**First contact**: Access to the primary health center through public transport.	It measures in four criteria, travel times, existence of universal public transport, accessible whereabouts, inventory of routes to reach the health center, existence of ramps and their characteristics.
	**Second contact**: Infrastructure that facilitates and ensures the free movement of the people with disabilities. The wheelchair is taken as a reference, under the idea that if a wheelchair circulates in a space, anyone with another technical aid can access it.	It measures on 6 criteria and 13 sub-criteria; exclusive parking for people with disabilities, ramps, handrails, access doors and access areas, floors, among others.
	**Third contact**: Until you reach the place destined for customer service, accessibility chain.	It measures on five criteria and seven sub-criteria; shift height, universal public guidance service, corridors, circulation areas and waiting rooms, among others.
Architectural Aspects	Implementation of universal design in the rest of the dependencies of the health center.	It measures in 3 criteria and 14 sub criteria; universal bathroom, door handles, floors, corridors, stairs, signage, among others.

**Table 2:** Relative frequency of average compliance of PHC centers according to accessibility dimensions.

N°	Accessibility Dimensions	% Compliance
1	Participation	37.7
2	Information	4.0
3	First Access Chain	43.1
4	Second Access Chain	37.0
5	Third Access Chain	53.2
6	Architectural Aspects	63.9
$\bar{x}$	Average fulfillment of all accessibility dimensions	39.8

## Data Availability

Not applicable.

## Abbreviations

The following abbreviations are used in this manuscript:

CESFAM	Family Healthcare Center
DALCO	Ambulation, Apprehension, Location and Communication
LCL	Lower Control Limit
MAIS	Comprehensive Health Care Model
PHC	Primary Healthcare
SOME	Medical and Statistic Orientation Service
UN	United Nations
WHO	World Health Organization
